# Enhanced Integrin Activation of PLD2-Deficient Platelets Accelerates Inflammation after Myocardial Infarction

**DOI:** 10.3390/ijms21093210

**Published:** 2020-05-01

**Authors:** Aglaia Maria Klose, Meike Klier, Simone Gorressen, Margitta Elvers

**Affiliations:** 1 Department of Vascular and Endovascular Surgery, Experimental Vascular Medicine, Heinrich-Heine University Medical Center, 40225 Düsseldorf, Germany; Aglaia-Maria.Klose@uni-duesseldorf.de (A.M.K.); meike.klier@med.uni-duesseldorf.de (M.K.); 2 Institute for Pharmacology and Clinical Pharmacology, Heinrich-Heine University, 40225 Düsseldorf, Germany; Simone.Gorressen@med.uni-duesseldorf.de

**Keywords:** Phospholipase D2, myocardial infarction, inflammation, integrin, Interleukin-6, TGF-β, TNF-α

## Abstract

Background: Phospholipase (PL)D1 is crucial for integrin α_IIb_β_3_ activation of platelets in arterial thrombosis and TNF-α-mediated inflammation and TGF-β-mediated collagen scar formation after myocardial infarction (MI) in mice. Enzymatic activity of PLD is not responsible for PLD-mediated TNF-α signaling and myocardial healing. The impact of PLD2 in ischemia reperfusion injury is unknown. Methods: PLD2-deficient mice underwent myocardial ischemia and reperfusion (I/R). Results: Enhanced integrin α_IIb_β_3_ activation of platelets resulted in elevated interleukin (IL)-6 release from endothelial cells in vitro and enhanced IL-6 plasma levels after MI in PLD2-deficient mice. This was accompanied by enhanced migration of inflammatory cells into the infarct border zone and reduced TGF-β plasma levels after 72 h that might account for enhanced inflammation in PLD2-deficient mice. In contrast to PLD1, TNF-α signaling, infarct size and cardiac function 24 h after I/R were not altered when PLD2 was deleted. Furthermore, TGF-β plasma levels, scar formation and heart function were comparable between PLD2-deficient and control mice 21 days post MI. Conclusions: The present study contributes to our understanding about the role of PLD isoforms and altered platelet signaling in the process of myocardial I/R injury.

## 1. Introduction

Phospholipase (PL)D1 and PLD2 belong to the family of phospholipases that catalyze the degradation of phosphatidylcholine into phosphatidic acid (PA) and choline. As a second messenger, PA plays a very important role in many cellular processes such as cell adhesion, cell migration and cell survival [1,2]. The two isoforms have a 50% homologous sequence [3] and are expressed in leucocytes and in platelets, where they are important for integrin-mediated cell adhesion [4]. PLD activity can be differentially regulated. PLD1 displays a very low basal activity and becomes activated by members of the Rho family, such as RhoA, Rac1 and the protein kinase C (PKC). In contrast, PLD2 exhibits a high basal activity, but the activation potential induced by different activators is very low [2].

In platelets, PLD1 plays an important role in glycoprotein (GP)Ib-mediated integrin activation and cell adhesion, and genetic deletion of PLD1 protects mice from arterial thrombosis and ischemic-dependent diseases like ischemic brain infarction [4]. Under inflammatory conditions, PLD1 supports α_IIb_β_3_ integrin activation and firm adhesion of platelets and immune cells to the injured endothelium. Loss of PLD2 does not alter integrin activation in healthy mice, although PLD activity in platelets was reduced in the absence of PLD2 [5]. In mice lacking both isoforms, defects in integrin activation and degranulation protected these mice against arterial thrombosis [6] and ischemic stroke [7]. The same results have been reported when platelets and mice were treated with the PLD inhibitor FIPI (5-Fluoro-2-Indolyl Deschlorohalopemide), which blocks the enzymatic activity of both PLD isoforms [7].

After cardiac ischemia and reperfusion (I/R) injury, loss of PLD1 led to increased infarct size and impaired left ventricular function 28 days after myocardial infarction (MI) compared to control mice. Reduced TNF-α-mediated inflammation in the acute phase after I/R and altered TGF-β mediated collagen scar formation was shown to be responsible for declined cardiac function [8]. Pharmacological inhibition of PLD showed that the enzymatic activity of PLD had no impact on TNF-α signaling and healing after MI in mice [9]. However, nothing is known about the impact of PLD2 on TNF-α regulation and myocardial healing after I/R in mice.

In this study, we were able to show that PLD-mediated regulation of TNF-α is restricted to PLD1 because loss of PLD2 did not impede TNF-α signaling, infarct size or cardiac function after iI/R. However, PLD2 deficiency led to enhanced platelet integrin activation in the very acute phase after MI. Altered integrin activation induced increased IL-6 release from endothelial cells in vitro and elevated IL-6 plasma levels after MI in vivo. This was accompanied by an enhanced inflammatory response including elevated migration of inflammatory cells into the infarct zone of the left ventricle 24 h after I/R.

## 2. Results

### 2.1. Loss of PLD2 Accelerates Inflammation after Myocardial Infarction

**Figure 1:**
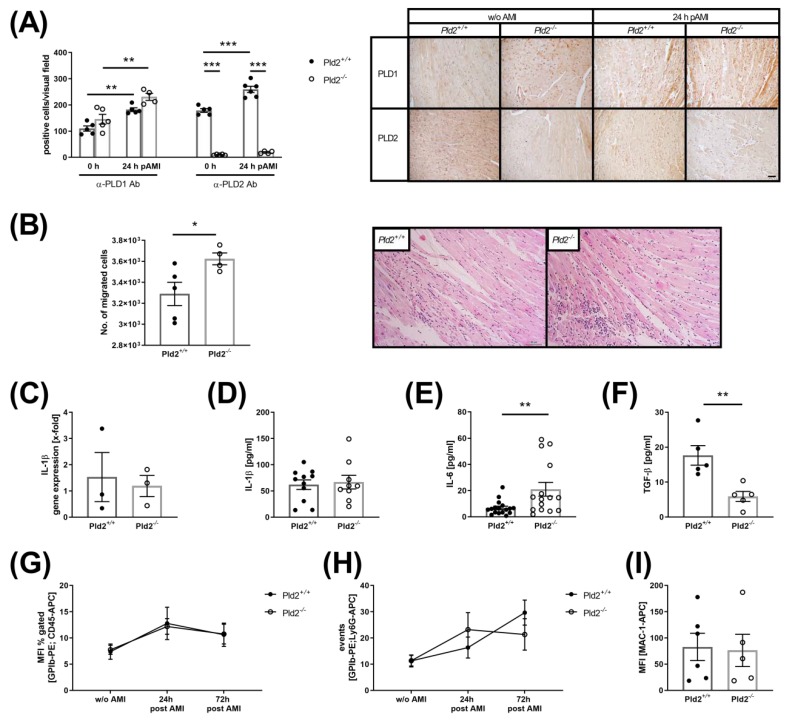
Increased inflammatory response in PLD2-deficient mice 24 h after I/R. (**A**) Increased PLD1 and PLD2 protein expression in cardiac sections of the left ventricle 24 h after I/R. Left: Determination of either PLD1- or PLD2-expressing cells. Positive cells werecounted. Right: Representative images of paraffin-embedded heart sections before and 24 h after I/R stained for either PLD1 or PLD2, *n* = 4-6, scale bar 50 µm. (**B**) Cardiac sections were stained with hematoxylin and eosin 24 h after I/R to analyze the migration of inflammatory cells into the infarct border zone. Left: Quantitative analysis of the migration of inflammatory cells. Right: Representative images are shown:, *n* = 4, scale bar = 50 μm. (**C**) Quantitative analysis of pro-inflammatory cytokine IL-1β in the left ventricle using real-time PCR, *n* = 3 and (**D**) in the plasma 24 h post I/R, n=9-11. (**E**) Quantitative analysis of IL-6 in the plasma of *Pld2^+/+^* and *Pld2^−/−^* mice 24 h after I/R, *n* = 15. (**F**) Quantitative analysis of TGF-β plasma levels 72 h after I/R, *n* = 5. (**G**) Flow cytometric analysis of platelet–leukocyte and (**H**) platelet–neutrophil aggregate formation in the plasma of healthy mice and 24 h and 72 h post I/R, *n* = 6. (**I**) Flow cytometric quantification of MAC-1 exposure on macrophages 24 h after I/R, *n* = 6. Bar graphs depict mean values ± SEM. Statistical analysis was performed using two-way ANOVA with Sidak’s post-hoc test (**A**) and a two-tailed Student’s *t*-test (**B**–**I**). * *p* < 0.05, ** *p* < 0.01 and *** *p* < 0.001. AMI = acute myocardial infarction.

Recent studies provide evidence that PLD1 plays a role in TNF-α-mediated inflammation and scar formation after MI [8]. To investigate if PLD2 has any impact on mycoardial healing and remodeling we ligated the left anterior descending artery (LAD) for 60 min and allowed reperfusion for 24 h after ischemia using PLD2-deficient and wild-type mice. To investigate PLD1 and PLD2 protein expression in the infarct border zone, we stained PLD1 and PLD2 positive cells in the left ventricle of PLD2-deficient and control mice, respectively (Figure 1A). While no alterations in PLD1 protein expression were detected between both groups, we found enhanced protein expression of both PLD isoforms in the infarct border zone compared to the left ventricle of healthy mice. However, real-time PCR analysis to quantify the relative mRNA expression of Pld2 in the left ventricle revealed significant downregulation of *Pld2* 24 h after MI compared to healthy control mice (Supplementary Materials, Figure 1A). Next, cardiac sections were analyzed for the migration of inflammatory cells into the infarct border zone (Figure 1B). Hematoxylin/eosin staining revealed enhanced migration of inflammatory cells into the left ventricle 24 h after ischemia. The expression and plasma levels of the acute phase cytokine IL-1β were not altered (Figure 1C,D). In contrast, PLD2-deficient mice exhibited enhanced IL-6 plasma levels 24 h (Figure 1E) and decreased TGF-β levels 72 h after MI (Figure 1F). Flow cytometric analysis of the formation of platelet–leukocyte and platelet–neutrophil conjugates as well as MAC-1 expression on neutrophils revealed no alterations between PLD2-deficient and control mice (Figure 1G–I).

### 2.2. Enhanced Activation of Platelet Integrin α_IIb_β_3_ in the Acute Phase after Cardiac Ischemia is Responsible for Enhanced IL-6 Release from Endothelial Cells

**Figure 2:**
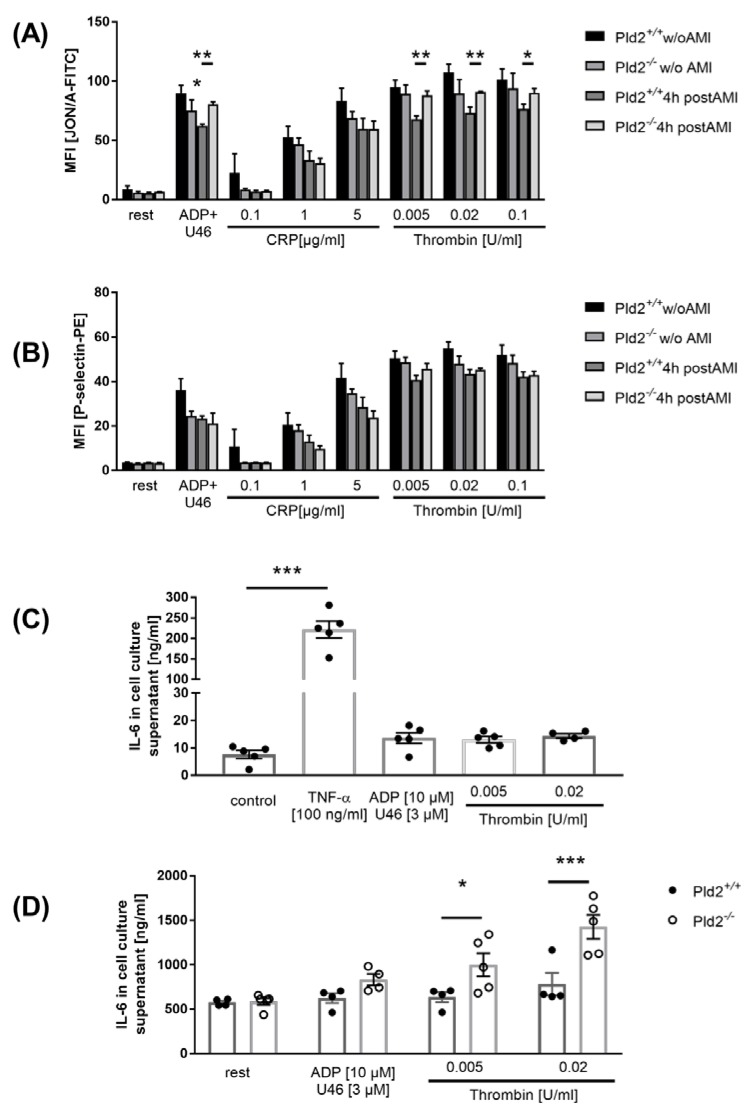
Platelet activation and platelet-induced IL-6 release of endothelial cells were determined. (**A**,**B**) Murine platelets were isolated 4 h after I/R and incubated with classical agonists. (**A**) Integrin a_IIb_β_3_ activity and (**B**) P-selectin expression were measured by flow cytometry, *n* = 5. (**C,D**) Quantitative analysis of IL-6 release of the endothelia cell line MHEC5-T stimulated with either 100 ng/mL TNF-α (positive control) or 10 µM ADP, 3 µM U46619, 0.005 and 0.02 U/mL thrombin in the absence (**C**) or presence (**D**) of platelets. Statistical analysis was performed using a two-tailed Student’s *t*-test (**A,B**) and a one-way ANOVA with Dunnett’s post-hoc test (**C**) or a two-way ANOVA with Sidak’s post-hoc test (**D**), *n* = 5. Bar graphs depict mean values ± SEM, * *p* < 0.05, ** *p* < 0.01, and *** *p* < 0.001. Rest = resting, ADP = adenosine diphosphate, CRP = collagen-related peptide, Thr = thrombin, U46619 = thromboxane analog. AMI = acute myocardial infarction.

We next measured platelet activation 4 h post MI using flow cytometry. Integrin α_IIb_β_3_ activation (JON/A binding integrin α_IIb_β_3_) and degranulation (P-selectin exposure) were determined (Figure 2A,B). In line with recent published data, no differences in platelet activation were detected in healthy PLD2-deficient compared to wild-type mice [6]. However, 4 h post MI, increased integrin activation in response to G-protein coupled receptor activation, but no alterations in degranulation, were observed in platelets from PLD2-deficient mice (Figure 2A,B).

Integrin α_IIb_β_3_ is important for platelet adhesion to endothelial cells upon inflammation [10]. Since PLD1 in platelets is able to trigger the release of IL-6 from endothelial cells after co-incubation of both cell types [5], we analyzed IL-6 release from endothelial cells (MHEC5-T) that were co-incubated with platelets from PLD2-deficient and control mice. In line with enhanced IL-6 plasma levels in PLD2-deficient mice 24 h after MI, increased secretion of IL-6 from endothelial cells was detected when incubated with PLD2-deficient platelets that were stimulated with G-protein coupled receptor agonists (Figure 2C,D).

### 2.3. PLD2 Does Not Regulate TNF-α Expression and Release

TNF-α is one of the acute phase cytokines involved in the inflammatory response after MI [11]. PLD1 plays a crucial role in TNF-α-mediated inflammation and scar formation after MI because PLD1 regulates TNF-α expression and release upon inflammation via phosphorylation of MEK1/2 and ERK1/2 [8,12].

**Figure 3:**
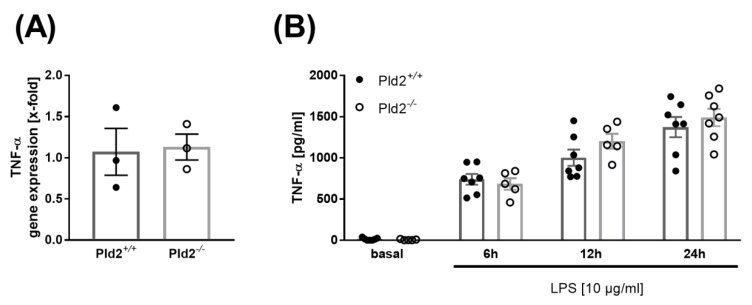
PLD2 does not regulate TNF-α expression and release. (**A**) TNF-α expression in the left ventricle of *Pld2^+/+^* and *Pld2^−/−^* mice 24 h after I/R was quantified by quantitative RT-PCR, *n* = 3. (**B**) Monocytes from whole blood of *Pld2^+/+^* and *Pld2^−/−^* mice were isolated and stimulated with lipopolysaccharide (LPS) to analyze TNF-α release at different time points, *n* = 3. Bar graphs depict mean values ± SEM. Statistical analysis was performed using two-tailed Student’s *t*-test.

In contrast to PLD1-deficient mice, no alterations in the expression of TNF-α were observed in mice that lack PLD2 24 h after MI (Figure 3A). This result was confirmed by in vitro analysis of monocytes that were isolated from whole blood of PLD2-deficient and control mice. Monocytes were stimulated with lipopolysaccharide (LPS) and analyzed for the release of TNF-α into the supernatant. Again, no alterations in TNF-α levels between PLD2-deficient and control monocytes were observed (Figure 3B).

### 2.4. Unaltered Infarct Size and Cardiac Function in PLD2-Deficient Mice 24 h after Ischemia and Reperfusion

**Figure 4:**
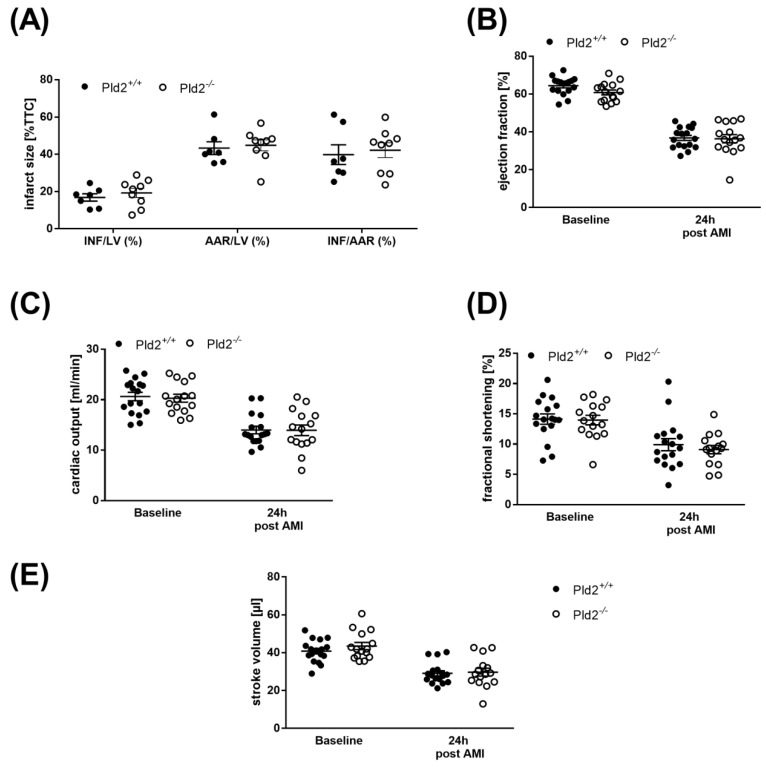
Unaltered infarct size and cardiac function in PLD2-deficient mice 24 h after I/R. (**A**) Determination of infarct size and area at risk (AAR) as percentage of the left ventricle (LV), and infarct size (INF) as percentage of the area at risk showed no differences as analyzed by TTC staining 24 h after I/R in *Pld2^+/+^* vs. *Pld2^-/^*^-^ mice, *n* = 7–9. (**B–E**) Echocardiographic analysis (baseline versus 24 h after I/R) of (**B**) ejection fraction, (**C**) cardiac output, (**D**) fractional shortening and (**E**) stroke volume, *n* = 6. Bar graphs depict mean values ± SEM. Statistical analysis was performed using the two-tailed Student’s *t*-test. AMI = acute myocardial infarction.

24 h after I/R, infarct size was assessed by 2,3,5-triphenyltetrazolium chloride (TTC) staining to differentiate between metabolically active and inactive tissue. Determination of infarct areas revealed no differences between PLD2-deficient and control mice (Figure 4A). Left ventricle function as shown by the analysis of ejection fraction, fractional shortening, cardiac output and stroke volume was then analyzed by echocardiography. As shown in Figure 4B–E, hemodynamic parameters were decreased in both PLD2-deficient and control mice, but no major alterations in cardiac function were detected between both groups (Figure 4B–E). Determination of the Spearman correlation between plasma levels of IL-6 and ejection fraction and between IL-6 and infarct size of Pld2^+/+^ and Pld2^−/−^ mice revealed a clear correlation between infarct size and IL-6 plasma levels in control mice (Spearman correlation coefficient (*ρ)* = 0.8255, *p* < 0.05) but not in PLD2-deficient mice (*ρ* = 0.189, *p* = 0.6262) (Supplementary Materials, Figure 2). No correlation between IL-6 plasma levels and ejection fraction was detected (*ρ* = 0.0053, *p* = 0.9836, control mice vs. *ρ* = 0.2253, *p* = 0.4016, PLD2-deficient mice) (Supplementary Figure S2).

### 2.5. Cell Survival is Not Altered in PLD2-Deficient Mice after Ischemia and Reperfusion Injury

**Figure 5:**
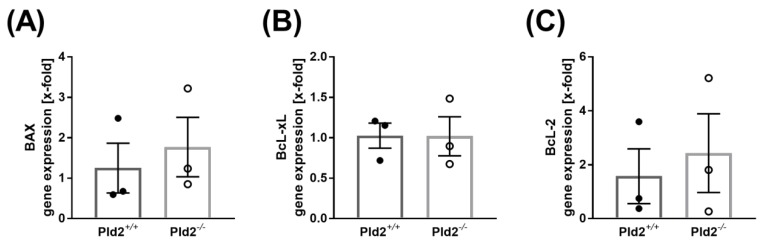
Expression of pro- and anti-apoptotic proteins after I/R. (**A**) Expression of the pro-apoptotic protein Bax and (**B**,**C**) the anti-apoptotic proteins Bcl-2 (**B**) and Bcl-xL (**C**) in the left ventricle of *Pld2^+/+^* and *Pld2^−/−^* mice 24 h after I/R, *n* = 3. Bar graphs depict mean values ± SEM. Statistical analysis was performed using a two-tailed Student’s *t*-test.

To determine any differences in cell survival, the expression of anti- and pro-apoptotic markers in the left ventricle of PLD2-deficient and control mice were analyzed. 24 h after MI, the expression of the pro-apoptotic marker Bax was not different between PLD2-deficient and control mice (Figure 5A). Bcl-2 and Bcl-xL are considered as anti-apoptotic markers [13] and were analyzed in the left ventricle as well. As shown in Figure 5B, C, no differences in the expression of Bcl-2 and Bcl-xL were measured when we used heart tissue of the left ventricle of PLD2-deficient and control mice (Figure 5B,C). However, additional experiments such as determination of caspase-3 activation in the left ventricle are needed to verify the role of PLD2 in cell survival and apoptosis after MI in near future.

### 2.6. Loss of PLD2 Does Not Modify Remodeling and Scar Formation 21 Days after Myocardial Infarction

**Figure 6:**
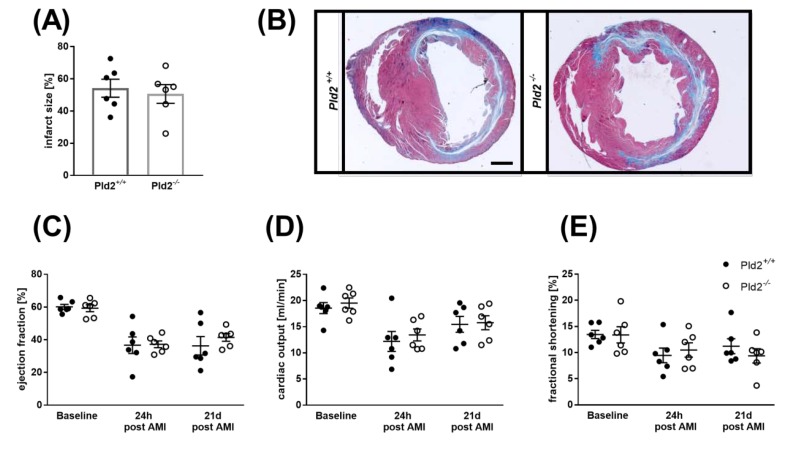

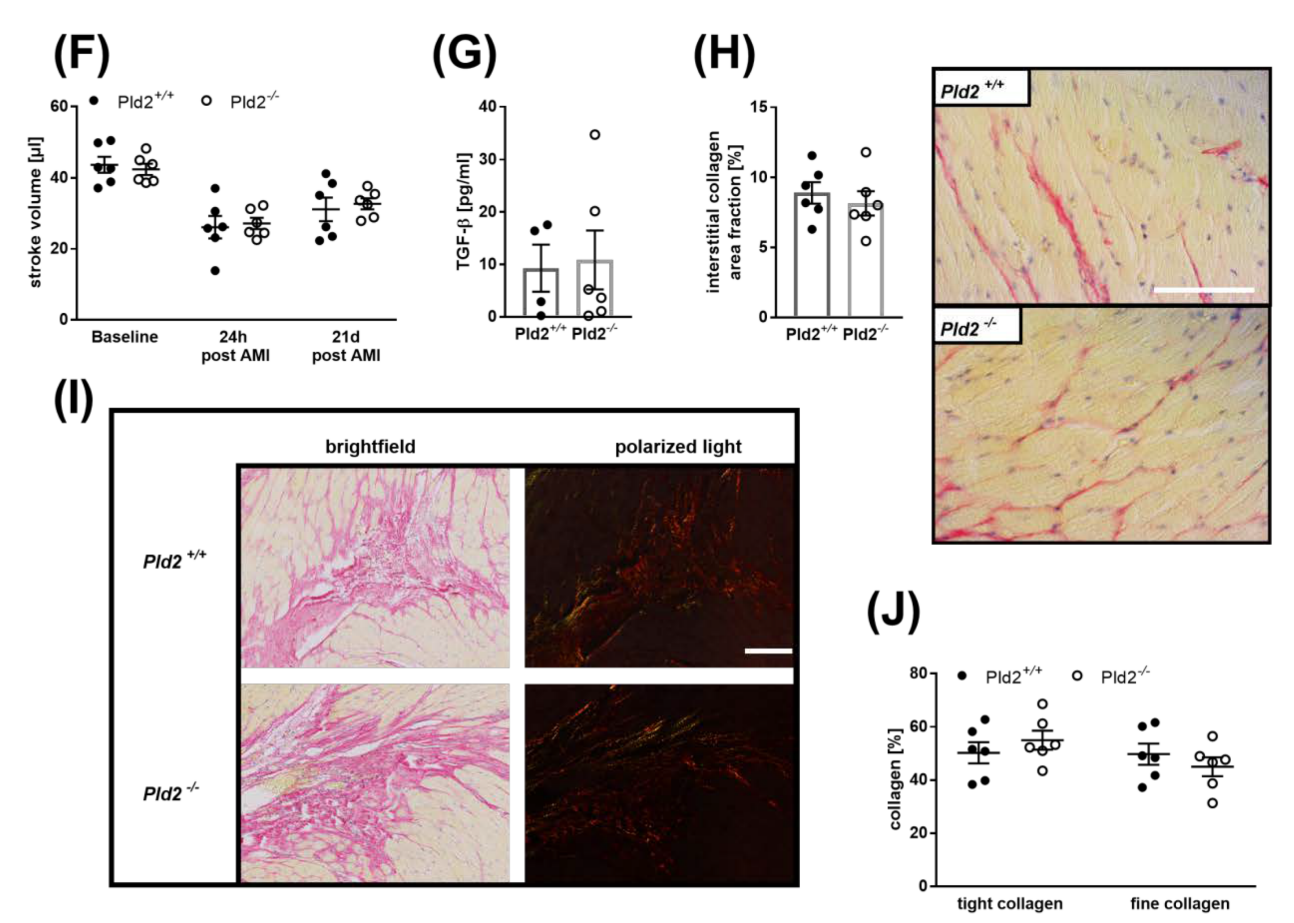
Unaltered scar formation and cardiac remodeling 21 d post MI in *Pld2^−/−^* mice. (**A**) Quantification of the infarct zone as percentage of the left ventricle in transversal cardiac sections stained with Gomori’s step one trichrome, *n* = 6. (**B**) Representative images are shown. Scale bar 1 mm. (**C–F**) Echocardiographic analysis (baseline versus 24 h and 21 days after I/R) of (**C**) ejection fraction, (**D**) cardiac output, (**E**) fractional shortening and (**F**) stroke volume, *n* = 6. (**G**) Quantitative analysis of TGF-β in plasma 21 days after I/R, *n* = 4–6. (**H–J**) Cardiac sections were stained with Sirius red 21 d after I/R to analyze (**H**) the amount of interstitial collagen at the infarct border zone and (**I–J**) the quality of collagen in the infarct zone of the left ventricle as marker of cardiac remodeling, *n* = 6. Representative images are shown (**H** and **I**, scale bar 100 µm). Bar graphs depict mean values ± SEM. Statistical analysis was performed using a two-tailed Student’s *t*-test. AMI = acute myocardial infarction.

To analyze the consequences of reduced inflammation 24 h post MI, we next investigated cardiac damage and repair 21 days post cardiac ischemia. Determination of infarct size by Gomori staining and cardiac function by echocardiography revealed no differences in infarct size (Figure 6A,B), ejection fraction, cardiac output, fractional shortening or stroke volume (Figure 6C–F) between PLD2-deficient and control mice. To analyze matrix remodeling after MI in detail, we first analyzed plasma levels of TGF-β at late stage of MI. TGF-β plasma levels were comparable between PLD2-deficient and control mice as detected 21 days after MI (Figure 6G). TGF-β promotes myofibroblast conversion from heart resident fibroblasts and extracellular matrix synthesis in healing hearts post MI [14]. Therefore, we investigated collagen deposition and organization in further detail. Quantification of interstitial fibrosis as determined by Sirius red staining of the remote myocardium revealed no major differences between PLD2-deficient and control mice (Figure 6H). To analyze collagen organization, we investigated fibrillar collagen density 21 days post MI. Birefringence analysis of Sirius red staining revealed no differences between tightly packed and fine collagen fibrils between mice with genetic deletion of PLD2 and controls (Figure 6I,J).

## 3. Discussion

The present study revealed that PLD2 regulates inflammation by platelet-induced elevation of IL-6 release from endothelial cells in the acute phase after myocardial I/R. Increased inflammation resulted in enhanced migration of inflammatory cells into the infarct border zone in the left ventricle. However, altered inflammatory responses of PLD2-deficient mice did not cause differences in infarct size, cardiac damage, scar formation or left ventricular function after 24 h and 21 days post MI. In addition, PLD2 did not modify TNF-α expression and release as recently shown for PLD1 [8].

PLD activity is enhanced in hearts after I/R [15] and was reported to be involved in processes of ischemic preconditioning in rabbit hearts [16]. Increased PLD1 protein levels have been detected in the remote myocardium after MI but not in the scar that shows PLD2 protein expression [17]. Moreover, the generation of PA by enzymatic activity of PLD is important for heart function because of its ability to increase the intracellular concentration of free Ca^2+^ in adult cardiomyocytes to augment cardiac contractile activity of the normal heart. Furthermore, PA is considered as an important signal transducer in cardiac hypertrophy [18]. Over recent years we have provided evidence for PLD1 to modulate inflammation and scar formation, including the regulation of TNF-α expression and release leading to enhanced infarct size, reduced quality of the scar tissue and declined cardiac function 28 days post MI [8]. Using the PLD inhibitor FIPI that blocks the enzymatic activity of both isoforms PLD1 and PLD2 revealed that PLD1-mediated regulation of TNF-α, cardiac function and scar formation is dependent on the non-enzymatic properties of PLD, while the migration of inflammatory cells into the infarct border zone after MI depends on the lipase activity of PLD. This was due to reduced infiltration of leukocytes into damaged cardiac tissue in both PLD1-deficient and FIPI-treated mice. However, only PLD1 deficiency resulted in reduced TNF-α signaling and enhanced cardiac damage as FIPI treatment of mice did not alter infarct size or heart function compared to non-treated control mice [9].

In contrast to PLD1-deficient mice, loss of PLD2 resulted in an increased inflammatory response 24 h after MI. Different reports in the past suggested an important role for PLD2 in inflammation. In contrast to the study of Speranza and colleagues who showed that downregulation of PLD2 inhibits the ability of cells to undergo chemotaxis, a more recent publication provided evidence for increased recruitment of neutrophils and macrophages in PLD2-deficient mice and upon Pld2 gene expression knockdown in acute lung injury [19,20]. Thus, different experimental approaches or inflammatory scenarios might play a role. It is also conceivable that the type of inflammation (acute or chronic) plays a role in PLD2-mediated migration of leukocytes or the injured organs such as lungs or heart.

Increased inflammation did not result in altered infarct size, scar formation or cardiac function neither at early (24 h) nor at late (21 days) time points. This is in line with different observations demonstrating that altered inflammation, matrix remodeling and left ventricle collagen network formation all contribute to left ventricular dysfunction [21]. Thus, it is not surprising that enhanced inflammation in PLD2-deficient mice did not lead to altered infarct size or heart function. This conclusion is emphasized by the analysis of PLD1-deficient mice that develop altered cardiac damage and function as a result of differences in the inflammatory response and cardiac remodeling compared to control mice [8].

Our results provide evidence for PLD2 as negative regulator of inflammation after MI. PLD2 was already shown to negatively regulate blood pressure via inhibition of the endothelial nitric oxide synthase (eNOS) signaling pathway [22]. In septic animals, loss of PLD2 is accompanied by increased bactericidal activity and recruitment of neutrophils into the lung [23]. The first evidence for PLD2 as a negative regulator of platelet function was shown using PLD1-deficient mice that were treated with the PLD inhibitor FIPI [24]. However, PLD2-deficient platelets from healthy mice do not show any alterations, suggesting that these differences result from non-enzymatic and enzymatic properties of PLD isoforms. Here, we provide direct evidence that platelets from mice that underwent MI have a different activation profile than platelets from healthy mice (Figure 2). While PLD2-deficient platelets from healthy donor mice do not show major differences in integrin activation or P-selectin exposure following stimulation with different agonists, increased platelet activation in response to G-protein coupled receptor activation was observed in PLD2-deficient platelets compared to control platelets 4 h after MI. These results suggest that MI induces altered platelet responses that can be detected in the blood circulation of mice.

Platelet inhibition by aspirin and P2Y_12_ inhibitors is indispensable in patients with acute myocardial infarction. Beside their role in thrombosis, platelets are known to modulate inflammation, cell survival and organ repair, but their role after MI is not well defined. Recent publications suggest that platelets play a role in the acute phase after MI because anti-platelet interventions inhibit inflammatory cell recruitment into the infarcted myocardium [25]. Blockage of the platelet major collagen receptor GPVI reduced infarct size 24 h post MI [26]. Furthermore, GPVI is involved in platelet adhesion to activated endothelium, the expression of inflammatory cytokines and myocardial function after MI [27,28,29]. In this study, we provide the first evidence that IL-6 plasma levels are regulated, at least in part, by platelet-mediated activation of endothelial cells after MI. However, our data did not show any direct effects of PLD2-deficient platelets on leukocytes because the number of platelet–leukocyte conjugates and levels of inflammatory cytokines such as IL-1β and TNF-α were unaltered. Thus, enhanced migration of inflammatory cells might be due to altered endothelial-derived IL-6. However, we cannot exclude that different cells and mechanisms account for the increase of IL-6 in the plasma of PLD2-deficient mice. Nevertheless, our in vitro data clearly showed that enhanced IL-6 plasma levels might be due, at least in part, to platelet–endothelial cell interactions that are modulated by platelet-derived PLD2.

In PLD2-deficient mice, TGF-β plasma levels were reduced at early time points after MI but normalize after 21 d compared to control mice (Figure 1F and Figure 6G). It is believed that platelets may be the main source of TGF-β in the early stage of infarct healing [14]. Thus, reduced TGF-β plasma levels after 72 h might be due to altered platelet activation in PLD2-deficient mice that underwent experimental MI. This reduction in TGF-β might account for enhanced inflammation in the acute phase after MI in PLD2-deficient mice because TGF-β regulates inflammatory leukocyte function by inhibiting macrophage pro-inflammatory gene expression. In contrast, macrophages and fibroblasts may be responsible for the sustained upregulation of TGF-β during the proliferative phase to promote myofibroblast conversion and extracellular matrix synthesis in healing infarcts [14,30]. At these later time points, no differences in TGF-β plasma levels were observed, suggesting that reduced TGF-β at early time points is the result of altered platelet activation in PLD2-deficient mice.

Taken together, this study adds new information about the impact of altered platelet activation on inflammation and uncovers the impact of PLD2 on inflammation and myocardial healing after MI to complement our understanding about the role of PLD isoforms in the process of cardiac damage.

## 4. Materials and Methods

### 4.1. Animals

Animal studies were performed in accordance with the guidelines of the European Parliament for the use of living animals in scientific studies and in accordance with the German law for protection of animals. The protocol was approved by Heinrich-Heine-University Animal Care Committee and by the district government of North-Rhine-Westphalia (LANUV; NRW; Permit Number 84-02.04.2015.A558, period 2016–2021). Gene targeted mice lacking PLD2 (*Pld2^−/−^*) were described previously [31]. The corresponding wild-type littermates were bred from breeder pairs and genotyped by PCR. Mice were anesthetised with Ketamin (Ketaset, Zoetis, Berlin, Germany, 100 mg/mL) and Xylazin (Wirtschaftsgenossenschaft deutscher Tierärzte eG (WDT), Garbsen, Germany, 20 mg/mL) by intraperitoneal (i.p.) injection before surgery. Euthanasia wasperformed by cervical dislocation.

### 4.2. Myocardial Ischemia and Reperfusion in Mice

For the analysis of MI, 10- to 12-week-old male mice were anesthetized by intraperitoneal injection of a solution with Ketamin (90 mg/kg body weight) and Xylazin (10 mg/kg body weight). MI was induced by ligation of the left anterior descending artery (LAD) for 60 min. Then, 24 h after reperfusion, the ischemic area (area of risk) and the infarcted area (infarct size) were determined by staining with TTC/Evans Blue solution. The ratios of the different areas were quantified digitally by video planimetry. For the analysis of infarct size in chronic series, infarct size was determined by Gomori’s one step trichrome staining 3 weeks after reperfusion. After progressing successfully with anesthesia, hearts were removed, fixed in 4% formalin, embedded in paraffin and cut in serial sections for staining with Gomori’s one step trichome solution. The infarct size was expressed as the percentage of the total left ventricular (LV) area. Additionally, echocardiography was performed at different time points using Vevo 2100 ultrasound machine (VisualSonics Inc., Bothell, Washington, USA) and different parameters, e.g., ejection fraction (%), cardiac output (mL/min), fractional shortening (%) and stroke volume (µL) were determined with corresponding software.

### 4.3. Collagen-Staining of Cardiac Sections

A total of 21 days after ischemia/reperfusion hearts were taken, they were embedded in paraffin and sections of these hearts were prepared. Scar formation was analyzed by staining of these sections with Gomori, Bouin’s (Sigma, Darmstadt, Germany) and hematoxylin solution (Carl Roth, Karlsruhe, Germany). Images were captured by Binocular Microscope (Nikon SMZ25, Tokyo, Japan), evaluated by Zen2 blue edition Software (Zeiss, Oberkochen, Germany) and the ratio of the infarct size to the total of the left ventricle was determined. To determine the amount of interstitial collagen, cardiac sections were stained by Picrosirius red staining (Morphisto, Frankfurt am Main, Germany) and Celestine blue solution (Sigma, Darmstadt, Germany) was used to stain the nuclei. Interstitial collagen was measured in percent by area fraction. Additionally, collagen density was analyzed by polarized light microscopy and evaluated by Image J software.

### 4.4. Immunhistochemistry of Cardiac Sections

24 h after cardiac I/R in mice, hearts were taken and paraffin sections of these hearts were stained with Haematoxylin/Eosin (HE) solution (Carl Roth). The total number of cells migrated into the infarcted area of the heart were counted per visual field and data are shown per 10^3^/mm^2^.

For the analysis of either PLD1- or PLD2-expressing cells in the left ventricle, streptavidin-biotin-immunoperoxidase staining of cardiac sections of paraffin-embedded hearts before and 24 h after ischemia/reperfusion was performed using rabbit anti mouse PLD1 antibody (Cell Signaling, Danvers, Massachusetts, USA) and rabbit anti mouse PLD2 antibody (Acris, Rockville, MD, USA), using a horseradish peroxidase (HRP)-labeled second antibody kit (LSAB2 System-HRP; DAKO, Santa Clara, California, USA) and Diaminobenzidine (DAB) reagent (DAKO) as chromogen. Positive cells were counted and data are presented per visual field.

### 4.5. Enzyme-Linked Immunosorbent Assay (ELISA)

For quantification of IL-1β, IL-6, TNF-α and TGF-β in plasma 24 h after ischemia/reperfusion, heparinized blood was centrifuged 10 min at 650 g. The plasma was taken and the cytokine amount was measured by Enzyme-Linked Immunosorbent Assay (ELISA) following the manufacturer’s protocol (DuoSet Mouse IL-1β/IL-1F2/ DuoSet Mouse IL-6/ DuoSet Mouse TNF-α, R&D Systems, Minneapolis, MN, USA). TGF-β in plasma of *Pld2^+/+^* and *Pld2^−/−^* mice was quantified 72 h and 21 days after I/R (DuoSet Mouse TGF-β, R&D Systems).

For analyzing the same cytokine levels in the supernatant of monocytes, monocytes were isolated, stimulated with 10 mg/µl lipopolysaccharide (LPS) and a supernatant was taken 0, 6, 12 and 24 h after stimulation. Murine monocytes were freshly isolated from whole blood of *Pld2^+/+^* and *Pld2^−/−^* mice. Four hundred ml of whole blood was centrifuged for 20 min at room temperature. Lymphocytes were separated using Biocoll Separating solution (Biochrom) with centrifugation for additional 10 min. Lysis of erythrocytes was followed by cell labeling using 10 mL CD11b Micro Beads (Miltenyi Biotec, Bergisch Gladbach, Germany) per 10^7^ cells. Monocytes were separated with a Vario Macs Separator (Miltenyi Biotec).

To investigate cytokine release of endothelia cells, IL-6 levels were measured in the supernatant of either via agonists (100 ng/mL TNF-α, 10 µM ADP (Sigma, Darmstadt, Germany), 3 µM U46619 (Tocris, Bristol, UK), 0.005 and 0.02 U/mL thrombin) stimulated MHEC5-T cells or after co-incubation with platelets pre-activated by the same agonists after 3.5 h. Because the entire platelet preparation was used, control experiments were performed to exclude endothelial cells secreting IL-6 in response to the platelet agonists ADP/U46619 or thrombin alone. MHEC5-T cells were co-incubated with ADP/U46619 or thrombin in the absence of platelets to provide evidence that these platelet agonists are not able to induce IL-6 release from endothelial cells per se.

All enzyme-linked immunosorbent assays were performed following the manufacturer’s protocol.

### 4.6. Flow Cytometric Analysis of Platelet-Immune Cell Aggregate Formation, Neutrophil and Platelet Activation

Before and 24 and 72 h after I/R, the formation of platelet-immune cell aggregates was measured via fluorescence based flow cytometry. Heparinized blood from *Pld2^+/+^* and *Pld2^−/−^* mice before and after I/R was washed twice with Tyrode’s buffer, centrifuged 5 min at 650× *g*, the supernatant was removed and only the cell-rich pellet was used for measurements. Samples were incubated with conjugated antibodies for platelets (GPIb- PE, Emfret, Eibelstadt, Germany) and either neutrophiles (Ly6G-APC, Biolegend, San Diego, California, USA) or leucocytes (CD45-APC, BD Bioscience, Heidelberg, Germany) and the MFI (mean fluorescence intensity) was determined.

To determine neutrophil activation before and 24 h after I/R, heparinized blood from *Pld2^+/+^* and *Pld2^−/−^* mice was washed twice with Tyrode’s buffer by centrifuging for 5 min at 650× *g*. The pellet was incubated with Ly-6G Dylight 488 (BD) and APC-CD11b (MAC-1) antibody for 15 min at room temperature. The MFI is representing MAC-1 exposure of activated neutrophils.

For measuring platelet activation, heparinized blood from *Pld2^+/+^* and *Pld2^−/−^* mice 0, 4, 24 h and 21 days after I/R was washed with Tyrode’s Buffer and centrifuged for 5 min at 650 g, the supernatant was removed and the pellet was diluted in Tyrode’s buffer for measurements. Samples were incubated with FITC-conjugated antibody against P-selectin (Emfret), PE-conjugated antibody against integrin α_IIb_β_3_ (Emfret) and classical agonists for platelet activation such as ADP, U46619, CRP (University of Cambridge, UK), or thrombin (Roche) for 7 min at 37 °C and 7 min at room temperature. Integrin activation and P-selectin exposure were determined by fluorescence-based flow cytometry.

### 4.7. Quantitative Real-Time PCR

For the analysis of endogenously expressed levels of Pld2, TNF-α, IL--1β, Bax and Bcl-xL only isolated total RNA of the left ventricle of the heart 24 h after ischemia of *Pld2^+/+^* and *Pld2^−/−^* mice was used. RNA isolation was performed by ReliaPrep RNA Tissue Miniprep System (Promega, Mannheim, Germany) following the manufacturer’s protocol. Quantitative real-time PCR was performed using Fast Sybr Green Master Mix (Thermo Fischer Scientific) following the manufacturer’s protocol. The expression level of the target was normalized to glyceraldehyde-3-phosphate dehydrogenase (Gapdh) RNA expression levels as a control. After reverse transcription, quantitative PCR amplification was performed using the following oligonucleotide primers: Pld2 for 5´GAAAGGGATAGGAAAGTCCAGG´3, rev 5´GGGTGGAAAGAGAACCCATAG´3; TNF–α for 5´GCCCCCATCTGACCCC-TTT´3; rev 5´GGGGCTGGCTCTGTGAGGAA´3; IL-1β for 5´AGCTTCCTTGTGCAAGTGTCTGAG´3, rev 5´TGTTGATGTGCTGCTGCGAGAT´3; Bax for 5´TGAAGACAGG GGCCTTTTTG´3; rev 5´AATTCGCCGGAGACACTCG´3; Bcl–xL for 5´GACAAGGAGATGCAGGTATTGG´3; rev 5´TCCCGTAGAGACCACAAAAGT´3; GAPDH for 5´GGTGAAGGCGGTG-TGAACG´3, rev 5´CTCGCTCCTGGAAGATGGTG´3.

### 4.8. Statistical Analysis

All experiments were performed at least three times with *n* defined as the individual animal. Data are presented as means ± SEM as indicated. Statistical analysis was performed using the two-tailed Student’s *t*-test, the one-way ANOVA with Dunnett’s post-hoc test or the two-way ANOVA with Sidak’s post-hoc test as indicated. A *p* value < 0.05 was set as significant. For all figures * *p* < 0.05, ** *p* < 0.01, and *** *p* < 0.001. Spearman correlation coefficient (*ρ*) was determined as indicated. *ρ* = −1 indicates a strong negative correlation, *ρ* = 0 indicates no correlation and *ρ* = +1 indicates a strong positive correlation.

## 5. Conclusions

PLD2-deficient platelets showed enhanced integrin activation at early time points after MI that is responsible for platelet-induced release of IL-6 from endothelial cells upon inflammation. In contrast to PLD1, PLD2 does not regulate TNF-α signaling, infarct size, scar formation or cardiac function after MI. Taken together, the present study contributes to our understanding about the role of PLD and altered platelet signaling in the process of myocardial I/R injury.

## Supplementary Materials

Supplementary materials can be found at https://www.mdpi.com/1422-0067/21/9/3210/s1.

## Abbreviations

AAR	Area at risk
ADP	Adenosine diphosphate
AMI	Acute myocardial infarction
CRP	Collagen-related peptide
ELISA	Enzym-Linked Immunosorbent Assay
FIPI	5-Fluoro-2-Indolyl Deschlorohalopemide
GAPDH	Glyceraldehyde-3-phosphate dehydrogenase
GP Ib/VI	Glycoprotein (GP) Ib/VI
HE	Hematoxylin/Eosin
I/R	Ischemia/reperfusion
IL-6	Interleukin 6
INF	Infarct
LAD	Left anterior descending artery
LPS	Lipopolysaccharide
LV	Left ventricle
Mac-1	Macrophage-1 antigen
MFI	Mean of fluorescence
MHEC5-T	Mouse Heart Endothelial Cell clone 5-Transformed
MI	Myocardial infarction
min	Minute
PA	Phosphatidic acid
PCR	(real-time) Polymerase Chain Reaction
PKC	Protein kinase C
PLD	Phospholipase D
rest	Resting
TGF-β	Transforming growth factor β
Thr	Thrombin
TNF-α	Tumor necrosis factor α
TTC	2,3,5-triphenyltetrazolium chloride
U46619	Thromboxane analog
