# Supplementary material for: Enhanced Integrin Activation of PLD2-Deficient Platelets Accelerates Inflammation after Myocardial Infarction
Source: Int J Mol Sci. 2020 May 1;21(9):3210. doi: 10.3390/ijms21093210 (PMC7247352; doi:10.3390/ijms21093210)
Supplement: Supplementary file 1 [file ijms-21-03210-s001.pdf]

International Journal of
Molecular Sciences

MDPI

Type of the Paper: Article

Enhanced integrin activation of PLD2 deficient platelets accelerates inflammation after myocardial infarction

Aglaia Maria Klose [1], Meike Klier [1], Simone Gorressen [2], and Margitta Elvers [1,*]

Supplemental Data

(A)

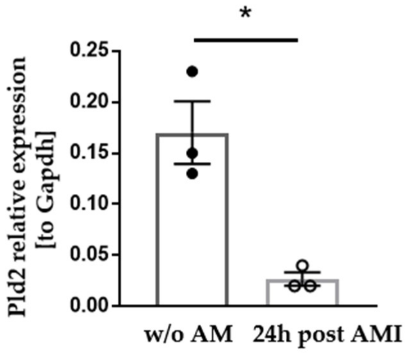

Suppl.-Figure S1. Quantification of mRNA expression of Pld2 in the left ventricle 24 hours after I/R injury in mice compared with healthy controls as determined by quantitative RT-PCR, n=3. **(A)** Bar graphs depict mean values ± SEM. Statistical analysis was performed by two-tailed Student's t-test. * $p < 0.05$. AMI = acute myocardial infarction.

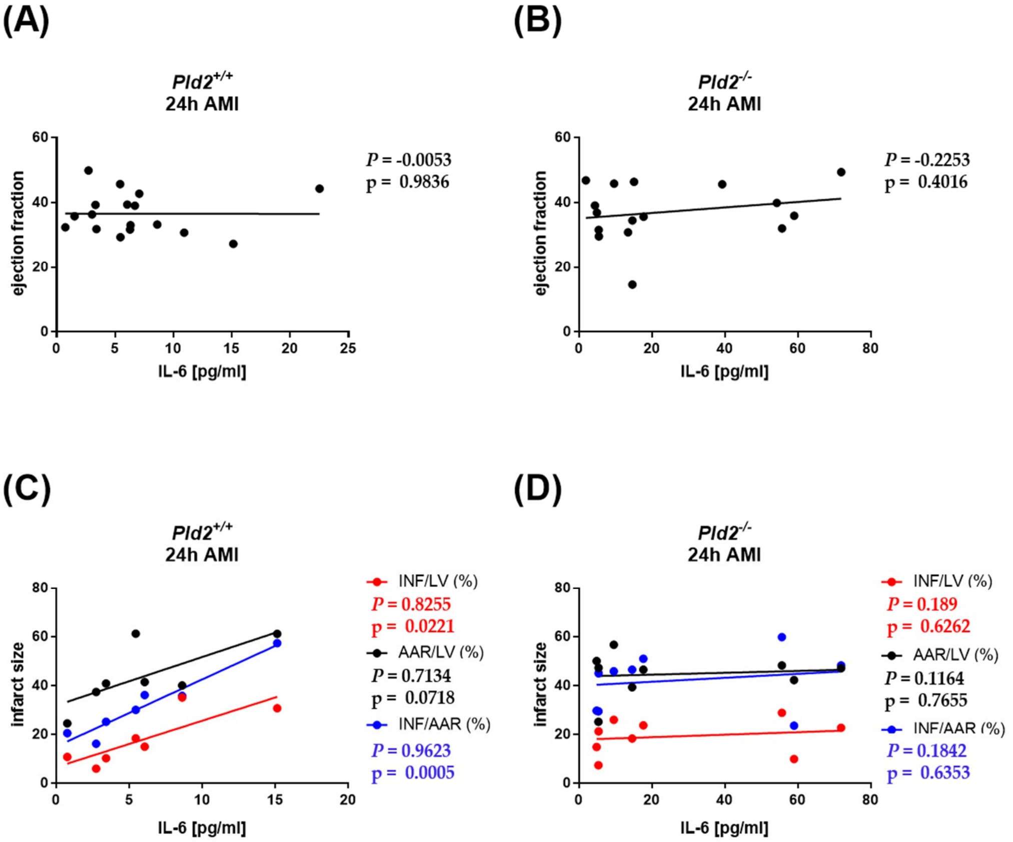

Suppl.-Figure S 2. Spearman correlation coefficient was determined as indicated. Spearman correlation coefficient (ρ) between plasma levels of IL-6 and ejection fraction **(A-B)** and IL-6 and infarct size **(C-D)** of *Pld2*$^{+/+}$ and *Pld2*$^{-/-}$ mice was determined. ρ = -1 indicates strong negative correlation, ρ = 0 indicates no correlation and ρ = +1 indicates strong positive correlation; pP-values as indicated. AMI = acute myocardial infarction.
